# Pimavanserin treatment increases plasma brain-derived neurotrophic factor levels in rats

**DOI:** 10.3389/fnins.2023.1237726

**Published:** 2023-08-30

**Authors:** Ashutosh Tripathi, Henry A. Nasrallah, Anilkumar Pillai

**Affiliations:** ^1^Faillace Department of Psychiatry and Behavioral Sciences, The University of Texas Health Science Center at Houston (UTHealth), Houston, TX, United States; ^2^Department of Psychiatry and Behavioral Neuroscience, University of Cincinnati, Cincinnati, OH, United States; ^3^Department of Psychiatry and Health Behavior, Augusta University, Augusta, GA, United States; ^4^Charlie Norwood VA Medical Center, Augusta, GA, United States

**Keywords:** Pimavanserin, Fluoxetine, BDNF, Parkinson’s Disease psychosis, neuroplasticity

## Abstract

**Background:**

Pimavanserin, a serotonin 5HT-2A receptor inverse agonist is the first-line, FDA-approved treatment of hallucinations and delusions associated with Parkinson’s Disease psychosis (PDP), which occurs in up to 50% of PD patients. The neurobiological mechanism underlying the therapeutic effectiveness of Pimavanserin in PDP remains unknown. Several earlier studies have shown that treatment with 5HT-2A antagonists and other drugs acting on the serotonergic system such as SSRIs increase Brain derived neurotrophic factor (BDNF) levels in rodents. BDNF is synthesized as the precursor proBDNF, that undergoes cleavage intra or extracellularly to produce a mature BDNF (mBDNF) protein. mBDNF is believed to play a key role in neuroplasticity and neurogenesis. The present study tested the hypothesis that treatment with Pimavanserin is associated with higher and sustained elevations of mBDNF.

**Methods:**

Adult Sprague–Dawley male rats were treated with Pimavanserin, Fluoxetine or vehicle for 4 weeks (chronic) or 2 h (acute). BDNF levels were determined by enzyme-linked Immunosorbent assay (ELISA).

**Results:**

We found significant increases in plasma mBDNF levels in rats following chronic Pimavanserin treatment, but not in Fluoxetine-treated rats. No significant changes in mBDNF levels were found in the prefrontal cortex or hippocampus following Pimavanserin or Fluoxetine treatment.

**Conclusion:**

These findings suggest that increase in mBDNF levels could be a contributing mechanism for the neuroprotective potential of Pimavanserin.

## Introduction

Parkinson’s disease (PD) is, by any measure, a neuropsychiatric disorder of major public health significance. It is the second most prevalent neurodegenerative disorder after Alzheimer’s Disease. Although treatment options for this disabling neuropsychiatric condition have increased (and improved) over time, it is sobering that the disabling motor symptoms of PD are further complicated by hallucinations and delusions in over 50% of patients (Aarsland et al., 1999; Eng and Welty, 2010; Forsaa et al., 2010). The emergence of these psychotic symptoms is associated with greater functional impairment, increased morbidity, increased nursing home placements, and accelerated mortality (Goetz and Stebbins, 1993; Marsh et al., 2004). There is ample and compelling evidence that the treatment of PD psychosis (PDP) with D2 antagonism, which is central to the mechanism of action of both typical and atypical antipsychotics can aggravate motor, cognitive and mood impairments in PD (Factor et al., 1995; Henderson and Mellers, 2000; Friedman, 2010). In addition, Quetiapine (seroquel) which is frequently used to treat PDP has failed to alleviate the symptoms of PDP in several randomized, small sample size-controlled trials (Ondo et al., 2005; Rabey et al., 2007; Shotbolt et al., 2009). On the other hand, Clozapine, the oldest atypical antipsychotic (Parkinson Study, 1999; The French Clozapine Parkinson Study, 1999) is associated with a potentially fatal risk of agranulocytosis, severe sedation, orthostatic hypotension (that leads to falls), obesity, diabetes, and hyperlipidemia, requiring frequent ongoing blood monitoring. Pimavanserin’s discovery and launch as the first evidence-based FDA-approved pharmacotherapy for PDP, circumvents all those complications from off-label therapies and has a placebo-like effect on the motor symptoms of PD because it is a potent, highly selective 5-hydroxytryptamine (serotonin) 2A receptor (5-HT2A) inverse agonist with no appreciable affinity to dopamine D2 receptors.

Pimavanserin displays nanomolar potency as a 5-HT2A receptor inverse agonist, selectivity for 5-HT2A over 5-HT2C receptors, and no meaningful activity at any other G-protein coupled receptor (Hacksell et al., 2014). In a Phase III clinical trial, Pimavanserin demonstrated strong improvements compared to placebo on the Scale for the Assessment of Positive Symptoms for Parkinson’s Disease Psychosis (SAPS-PD) scores, a scale adapted for use in Parkinson disease psychosis (PDP) (Meltzer et al., 2010). Also, improvements in all other efficacy endpoints, including physician’s clinical global impression, caregiver burden, night-time sleep quality and daytime wakefulness, were observed. Pimavanserin’s effects on the motor symptoms were similar to placebo and showed overall benign safety and tolerability in long-term use (Cummings et al., 2014). The above clinical studies are further supported by preclinical evidence that Pimavanserin, reversed the psychotic-like behavioral deficits in head twitch, amphetamine-induced hyperactivity and prepulse inhibition in 6-hydroxydopamine (6-OHDA) lesioned rats (McFarland et al., 2011). Together, these studies demonstrate that Pimavanserin offers an optimal treatment option for patients suffering from PDP.

The exact molecular mechanisms underlying the antipsychotic effects of Pimavanserin as an inverse agonist are still unknown. A number of earlier studies, including reports from our laboratory, have shown that treatment with 5HT-2A antagonists (which include pimavanserin) increase Brain derived neurotrophic factor (BDNF) levels in rodents and are neuroprotective (Vaidya et al., 1997; Pillai et al., 2006). BDNF is synthesized as a precursor, proBDNF, which is cleaved to the mature 14-kDa form (mBDNF) by protease tissue plasminogen activator (tPA)-mediated activation of plasmin (Lessmann et al., 2003). mBDNF signaling through its receptor, tropomyosin-related receptor kinase B (TrkB) has been shown to mediate adult neurogenesis and hippocampal long-term potentiation (LTP), a process involving persistent strengthening of synapses associated with learning and memory (Minichiello et al., 1999; Xu et al., 2000). On the other hand, proBDNF shows a high affinity for p75 neurotrophic receptor (p75NTR), which results in long-term depression (LTD) facilitation, and spine loss (Teng et al., 2005; Pillai, 2008; Kowianski et al., 2018). The signaling pathways of mBDNF and proBDNF are depend on each other and overall, they play opposite roles in the brain plasticity. Therefore, the mBDNF/proBDNF homeostasis is important for normal brain function whereas alterations in this balance could lead to neuronal death and impaired synaptic plasticity. Changes in the mBDNF to proBDNF ratio has been found in the plasma and cerebrospinal fluid (CSF) of a number of neuropsychiatric and neurological conditions (Zhou et al., 2013; Fleitas et al., 2018). This imbalance could be a result of abnormal enzymatic cleavage and conversion of proBDNF to mBDNF. BDNF is expressed throughout the adult mammalian brain including the prefrontal cortex (PFC) and hippocampus, two brain regions known to play central role in various cognitive and behavioral functions (Bramham and Messaoudi, 2005). In the present study, we tested the hypothesis that treatment with Pimavanserin is associated with higher and sustained elevations of mBDNF compared to vehicle. In addition to blood, we measured mBDNF and proBDNF levels in the PFC and hippocampus to investigate whether Pimavanserin treatment induces brain region-specific changes in mBDNF and proBDNF levels.

## Methods

### Animals

Male Sprague–Dawley rats, weighing 280–320 g (6–8 weeks old) were purchased from Charles River Laboratories, and were housed in the animal facility at Augusta University, GA, United States. Rats were housed and maintained (2 rats per cage) in standard polypropylene cages in a 12-h light–dark cycle in compliance with the US National Institute of Health guidelines, which was approved by Augusta University animal welfare guidelines.

### Drug treatment

Rats were administered with vehicle, Pimavanserin or Fluoxetine. Rats were divided in three groups (N = 10 per group). Group I -vehicle (0.9% saline; subcutaneous (s.c.) injection). Group II- Pimavanserin (1 mg/kg/d dissolved in 0.9% saline; s.c.). Pimavanserin was provided by Acadia Pharmaceuticals Inc. Group III-Fluoxetine (Sigma; 10 mg/kg/d dissolved in 0.9% saline; intraperitoneal (i.p.) injection). The Pimavanserin dose was selected based on previous studies in which the above dose has been shown to prevent disruption of pre-pulse inhibition induced by the 5-HT_2A_ agonist DOI (Vanover et al., 2006), and blocks stimulant-augmented locomotor activity in rat model of Parkinson’s disease (Halberstadt et al., 2013). Chronic fluoxetine treatment (10 mg/kg) induces an increase in BDNF levels in several brain regions in rats (Molteni et al., 2006; Larsen et al., 2008; Rogoz et al., 2008).

**Figure 1:**
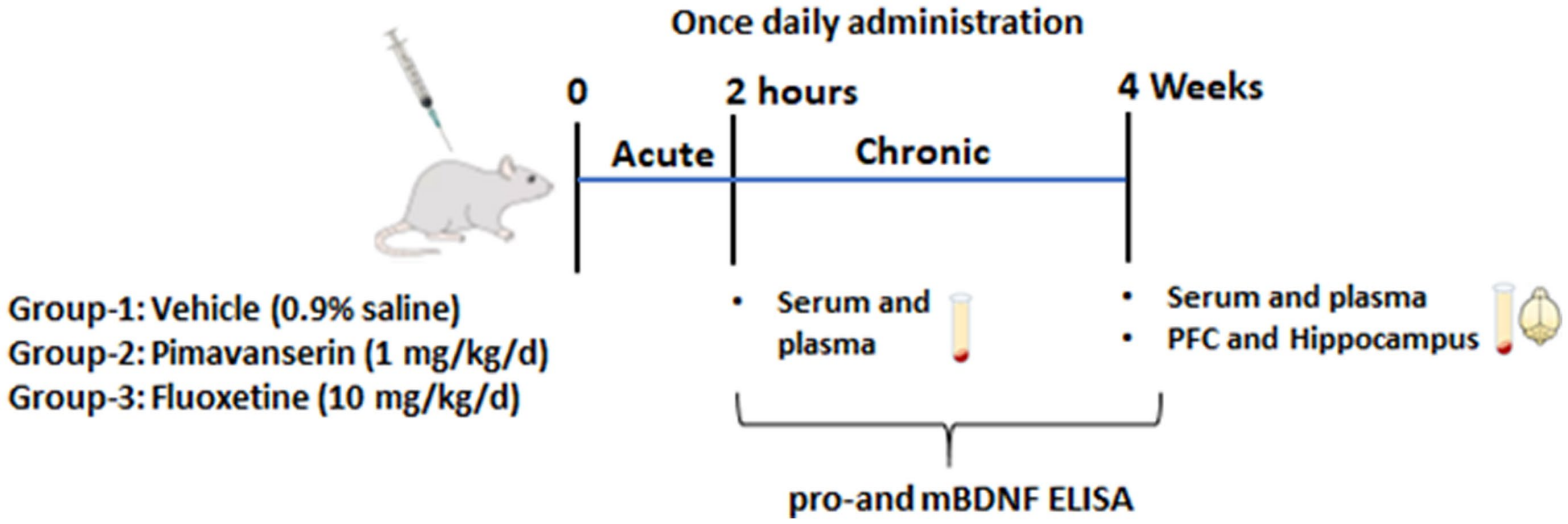
Schematic representation shows experimental plan. Male rats were divided in three groups (*N* = 10 per group). Group I -vehicle (0.9% saline). Group II- Pimavanserin (1 mg/kg/d dissolved in 0.9% saline) subcutaneous (s.c.) injections. Group III-Fluoxetine (10 mg/kg/d dissolved in 0.9% saline) intraperitoneal (i.p.) injection. Blood samples were collected via orbital sinus under anesthesia at 2 h following the first injection of vehicle or drug (acute treatment study). The same animals were continued with vehicle or drug treatment for 4 weeks, and animals were sacrificed under anesthesia to collect blood and brain tissues (chronic treatment study).

Same animals were used in acute and chronic treatment studies. Blood samples were collected via orbital sinus under anesthesia at 2 h following the first injection of vehicle or drug (acute treatment study). The same animals were continued with vehicle or drug treatment for 4 weeks, and animals were sacrificed under anesthesia to collect blood and brain tissues (chronic treatment study) (Figure 1).

### BDNF measurements

Rat plasma, serum, and brain (PFC and hippocampus) samples were collected immediately following decapitation under anesthesia using isoflurane. Whole hippocampus and the PFC tissues were individually dissected. Pro and mature forms of BDNF protein levels in samples were measured by enzyme-linked Immunosorbent assay (ELISA) according to the manufacturer’s instructions [ELISA Kit-Aviscera Bioscience, United States; proBDNF (# SK00752-06) and mBDNF (# SK00752-03B)]. Data were analyzed by One-way ANOVA followed by *post hoc*-Dunnett’s multiple comparisons test. The values are expressed as pg./ml for serum and plasma; and pg./ug of protein for the PFC and hippocampus samples.

## Results

Plasma BDNF reflect the BDNF from non-neuronal peripheral cells including vascular smooth muscle cells, endothelial cells, lymphocytes and monocytes (Donovan et al., 1995; Nakahashi et al., 2000), whereas serum BDNF levels largely reflect the amount of BDNF stored and released from platelets during coagulation. Plasma BDNF represents biologically active form or state-dependent marker (Serra-Millas, 2016; Polyakova et al., 2017). Evidence also suggests that plasma BDNF levels reflect brain BDNF levels (Karege et al., 2002; Pillai et al., 2010; Klein et al., 2011).

### Chronic treatment study

**Figure 2:**
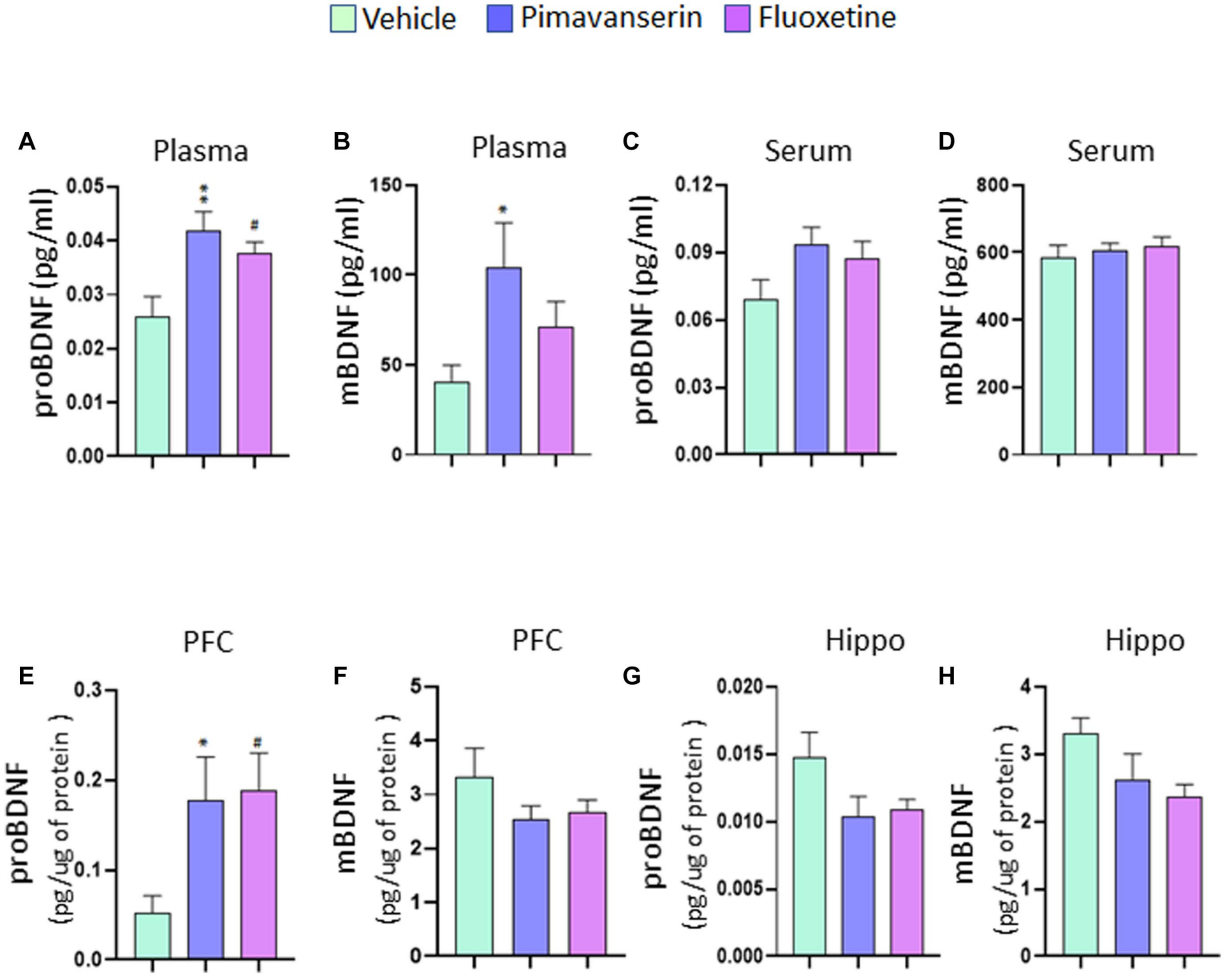
Chronic Pimavanserin treatment increased plasma mBDNF levels. Protein levels of mBDNF and proBDNF were examined in **(A,B)** plasma, **(C,D)** serum, **(E,F)** PFC and **(G,H)** hippocampus at 4 weeks following vehicle or drug administration by ELISA. **(A)** proBDNF protein levels in plasma. One-way ANOVA posthoc-Dunnett’s, treatment (*F* (2,27) = 6.558 *p* = 0.0048) **p < 0.01, #*p* < 0.05 vs. vehicle; *N* = 10. **(B)** mBDNF protein levels in plasma. One-way ANOVA posthoc-Dunnett’s, treatment (*F* (2,27) = 3.390 *p* = 0.0486) *p < 0.05 vs. vehicle; *N* = 10. **(C)** proBDNF protein levels in serum. One-way ANOVA posthoc-Dunnett’s, *N* = 10. **(D)** mBDNF protein levels in serum. One-way ANOVA posthoc-Dunnett’s, *N* = 10. **(E)** proBDNF protein levels in PFC. One-way ANOVA posthoc-Dunnett’s, treatment (*F* (2,27) = 4.497 *p* = 0.0206) *p < 0.05, #*p* < 0.05 vs. vehicle; *N* = 10. **(F)** mBDNF protein levels in PFC. One-way ANOVA posthoc-Dunnett’s, *N* = 10. **(G)** proBDNF protein levels in hippocampus. One-way ANOVA posthoc-Dunnett’s, *N* = 10. **(H)** mBDNF protein levels in hippocampus. One-way ANOVA posthoc-Dunnett’s, *N* = 10.

In chronic treatment studies, we found that both Pimavanserin and Fluoxetine significantly increased plasma levels of precursor form of BDNF (proBDNF) as compared to vehicle group (Figure 2A). A significant increase in plasma mature form of BDNF (mBDNF) was found in Pimavanserin-treated rats, but not in Fluoxetine-treated group (Figure 2B). No significant change in serum proBDNF and mBDNF levels was found in Pimavanserin or Fluoxetine-treated rats (Figures 2C,D). In addition, both Pimavanserin and Fluoxetine significantly increased proBDNF levels in the PFC as compared to vehicle group (Figure 2E). However, no significant changes in mBDNF levels in the PFC (Figure 2F), and mBDNF (Figure 2G) and proBDNF (Figure 2H) levels in the hippocampus were observed in any of the chronic treatment groups as compared to vehicle group.

### Acute treatment study

**Figure 3:**
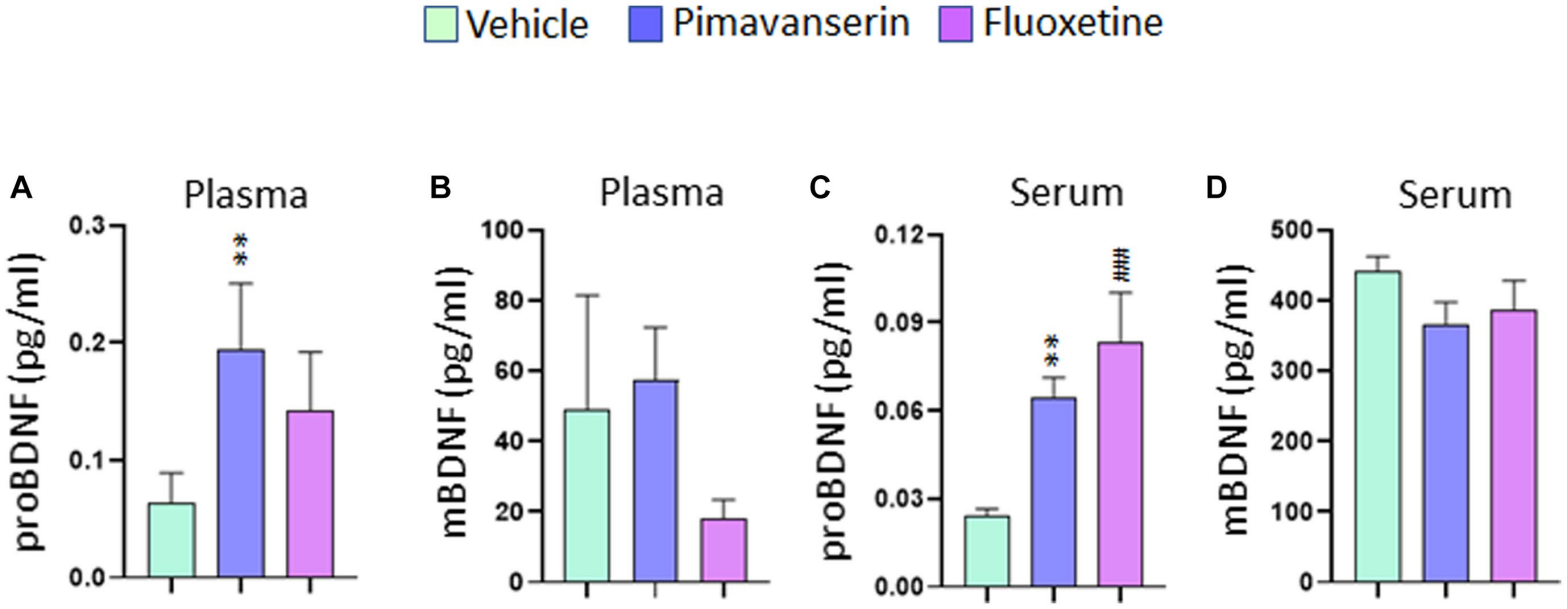
Acute Pimavanserin treatment increased plasma and serum proBDNF levels. Protein levels of mBDNF and proBDNF were examined in **(A,B)** plasma and **(C,D)** serum at 2 h following drug or vehicle administration by ELISA. **(A)** proBDNF protein levels in plasma. One-way ANOVA posthoc-Dunnett’s, treatment (*F* (2,27) = 4.860 *p* = 0.0157) **p < 0.01 vs. vehicle; *N* = 10. **(B)** mBDNF protein levels in plasma. One-way ANOVA posthoc-Dunnett’s, *N* = 10. **(C)** proBDNF protein levels in serum. One-way ANOVA posthoc-Dunnett’s, (*F* (2,27) = 10.91 *p* = 0.0003) **p < 0.01, ###*p* < 0.001 vs. vehicle; *N* = 10. **(D)** mBDNF protein levels in serum. One-way ANOVA posthoc-Dunnett’s, *N* = 10.

In acute treatment studies, Pimavanserin significantly increased proBDNF (Figure 3A), but not mBDNF (Figure 3B) levels in plasma and serum (Figures 3CD) as compared to vehicle group. Also, Fluoxetine treatment increased proBDNF levels in serum as compared to vehicle group (Figure 3C). However, no significant difference in serum or plasma mBDNF levels in any of the acute treatment groups as compared to vehicle group.

## Discussion

BDNF is highly expressed in the prefrontal cortex and hippocampus, brain areas known to regulate functions such as memory and emotion (Kapczinski et al., 2008). BDNF exists in two distinct forms, pro- and mature and exert opposite effects. proBDNF promotes neuronal cell death while mature BDNF promotes neuronal cell growth and survival (Pillai, 2008). A decrease in BDNF has been strongly associated with psychosis in a number of neuropsychiatric disorders (Buckley et al., 2007; Pillai, 2008). Plasma BDNF levels show significant negative correlation with positive symptoms at psychosis onset (Buckley et al., 2007). Furthermore, smaller hippocampal volumes in drug-naïve patients with first episode psychosis (and depression) are associated with lower BDNF levels (Rizos et al., 2011). Peripheral BDNF levels are significantly reduced in subjects with PD (Mogi and Nagatsu, 1999; Angelucci et al., 2016; Wang et al., 2016). Reductions in BDNF levels have also been reported in rodent models of PD (Hernandez-Chan et al., 2015; Johnson et al., 2015). Moreover, there is accumulating evidence of impaired synaptogenesis and defective neuronal plasticity in PD (Picconi et al., 2012). In addition, dysfunction of both dopaminergic and non-dopaminergic neurotransmission systems (serotoninergic, glutamatergic) has been documented in PD (Barone, 2010).

It is known that agonism of the 5HT2A receptor reduces the levels of BDNF whereas treatment with 5HT2A inverse agonists (i.e., potent antagonists) increases BDNF levels in rodents (Vaidya et al., 1997). Further, pimavanserin promotes BDNF and Glial cell line-derived neurotrophic factor (GDNF) release and protects primary dopaminergic neurons against 1-methyl-4-phenylpyridinium (MPP+) toxicity (Lavigne et al., 2021). 5HT2A blockade is also known to stimulate neurogenesis (Banasr et al., 2004). A recent study has shown that Pimavanserin reverses stressor-induced anxiogenic responses and normalizes plasma corticosterone levels in rodents (Malin et al., 2023). Stress and increased corticosterone levels have been shown to induce 5-HT2A receptor activity and expression. Pimavanserin suppresses both the constitutive and agonist-stimulated activity of the 5-HT2A receptor (Hacksell et al., 2014). Our study found significant increases in plasma mBDNF in chronic Pimavanserin-treated rats. Interestingly, no significant change in mBDNF was found in serum samples following Pimavanserin treatment.

Although BDNF exists in two different forms, many previous studies failed to differentiate between these two forms in the ELISA due to the limited specificity of the particular BDNF antibodies. In the present study, we were able to differentiate between mBDNF and proBDNF levels in our samples. We have exposed the animals to isoflurane in both acute and chronic treatment studies to collect tissue samples. Therefore, the effects of isoflurane on BDNF levels cannot be ruled out. However, a number of studies have reported inconsistent data on the influence of isoflurane on BDNF levels in rodents suggesting the need for additional studies to determine the dose and time-dependent effects of isoflurane on BDNF levels (Zhang et al., 2014; Antila et al., 2017; Zhang et al., 2019; Theilmann et al., 2020). It is important to note that we found significant changes in plasma, but not serum BDNF levels following chronic treatment with Fluoxetine or Pimavanserin. It is known that platelets are the major source of blood BDNF and platelets release BDNF during the clotting process (Fujimura et al., 2002; Lommatzsch et al., 2005). Evidence also suggest that serum BDNF levels reflect the amount of platelet-associated BDNF (Fujimura et al., 2002). On the other hand, plasma BDNF levels show a negative association with the fibrinogen level in patients with angina pectoris (Jiang et al., 2011). It is possible that BDNF in serum and plasma reflect two different pools of BDNF, and differently regulated by a number of factors including clotting time and centrifugation strategy (Gejl et al., 2019).

Although the mechanism underlying the observed drug-induced increases in proBDNF in the PFC is not known, this may be due, at least in part, to reduced proBDNF cleavage. It is well established that proBDNF cleavage depends on tPA/plasmin proteolytic activity (Pang et al., 2004; Barnes and Thomas, 2008; Tang et al., 2015; Wang et al., 2021). A significant decrease in tPA levels and/or an increase in plasminogen activator inhibitor type 1 (PAI-1) levels result in reduced proBDNF cleavage (Nagappan et al., 2009). Decreased plasma tPA levels have been reported in depressed subjects (Shi et al., 2010). Although Fluoxetine and Pimavanserin have different pharmacological profiles, it is possible that dysfunction of the tPA/PAI-1 system might represent a common pathophysiological mechanism shared by both Fluoxetine and Pimavanserin. Therefore, further studies are required to fully understand the role of tPA/PAI-1 pathway in mediating Pimavanserin-induced changes in BDNF levels and to determine whether the changes in this pathway are brain region-specific. The current study was conducted using one dose of Pimavanserin and therefore, additional studies are needed to examine whether the dose and duration of the treatment used in the study are sufficient for the conversion of proBDNF to mBDNF in the brain vs. periphery. Since the same animals were used in both acute and chronic treatment studies, we were able to collect only blood samples from the acute time point. It is important to determine the acute treatment effects of Pimavanserin on brain BDNF levels. Another limitation of the study is the lack of data from female rats. Therefore, future studies will investigate whether the effects of Pimavanserin on BDNF levels is sex-dependent.

In summary, we found a significant increase in plasma mBDNF levels in rats following chronic Pimavanserin treatment. Since plasma BDNF is capable of crossing the blood–brain barrier (Poduslo and Curran, 1996; Pan et al., 1998; Klein et al., 2011), changes in plasma mBDNF observed in our study might reflect changes in CNS BDNF levels. Together, these findings suggest that increase in mBDNF levels could be a contributing mechanism for the neuroprotective potential of Pimavanserin.

## Data availability statement

The original contributions presented in the study are included in the article/supplementary material, further inquiries can be directed to the corresponding author.

## Ethics statement

The animal study was approved by the Institutional Animal Care and Use Committee (IACUC), Augusta University. The study was conducted in accordance with the local legislation and institutional requirements.

## Author contributions

AP and HN designed the study. AT performed the analysis. AP wrote the manuscript draft. All authors contributed to the article and approved the submitted version.

## Funding

The study was supported by the Investigator Initiated Study funding from Acadia Pharmaceuticals Inc.

## Conflict of interest

The authors declare that this study received funding from Acadia Pharmaceuticals Inc.. The funder was not involved in the study design, collection, analysis, interpretation of data, the writing of this article, or the decision to submit it for publication.

## Publisher’s note

All claims expressed in this article are solely those of the authors and do not necessarily represent those of their affiliated organizations, or those of the publisher, the editors and the reviewers. Any product that may be evaluated in this article, or claim that may be made by its manufacturer, is not guaranteed or endorsed by the publisher.

## References

[ref1] Aarsland D. Larsen J. P. Cummings J. L. Laake K. (1999). Prevalence and clinical correlates of psychotic symptoms in Parkinson disease: a community-based study. Arch. Neurol. 56, 595–601. doi: 10.1001/archneur.56.5.595, PMID: 10328255

[ref2] Angelucci F. Piermaria J. Gelfo F. Shofany J. Tramontano M. Fiore M.. (2016). The effects of motor rehabilitation training on clinical symptoms and serum BDNF levels in Parkinson's disease subjects. Can. J. Physiol. Pharmacol. 94, 455–461. doi: 10.1139/cjpp-2015-0322 26863448

[ref3] Antila H. Ryazantseva M. Popova D. Sipilä P. Guirado R. Kohtala S.. (2017). Isoflurane produces antidepressant effects and induces TrkB signaling in rodents. Sci. Rep. 7:7811. doi: 10.1038/s41598-017-08166-9, PMID: 28798343 PMC5552878

[ref4] Banasr M. Hery M. Printemps R. Daszuta A. (2004). Serotonin-induced increases in adult cell proliferation and neurogenesis are mediated through different and common 5-HT receptor subtypes in the dentate gyrus and the subventricular zone. Neuropsychopharmacology 29, 450–460. doi: 10.1038/sj.npp.1300320, PMID: 14872203

[ref5] Barnes P. Thomas K. L. (2008). Proteolysis of proBDNF is a key regulator in the formation of memory. PLoS One 3:e3248. doi: 10.1371/journal.pone.0003248, PMID: 18813339 PMC2532744

[ref6] Barone P. (2010). Neurotransmission in Parkinson's disease: beyond dopamine. Eur. J. Neurol. 17, 364–376. doi: 10.1111/j.1468-1331.2009.02900.x, PMID: 20050885

[ref7] Bramham C. R. Messaoudi E. (2005). BDNF function in adult synaptic plasticity: the synaptic consolidation hypothesis. Prog. Neurobiol. 76, 99–125. doi: 10.1016/j.pneurobio.2005.06.003, PMID: 16099088

[ref8] Buckley P. F. Pillai A. Evans D. Stirewalt E. Mahadik S. (2007). Brain derived neurotropic factor in first-episode psychosis. Schizophr. Res. 91, 1–5. doi: 10.1016/j.schres.2006.12.026, PMID: 17306505 PMC1933504

[ref9] Cummings J. Isaacson S. Mills R. Williams H. Chi-Burris K. Corbett A.. (2014). Pimavanserin for patients with Parkinson's disease psychosis: a randomised, placebo-controlled phase 3 trial. Lancet 383, 533–540. doi: 10.1016/S0140-6736(13)62106-6, PMID: 24183563

[ref10] Donovan M. J. Miranda R. C. Kraemer R. McCaffrey T. A. Tessarollo L. Mahadeo D.. (1995). Neurotrophin and neurotrophin receptors in vascular smooth muscle cells. Regulation of expression in response to injury. Am. J. Pathol. 147, 309–324. PMID: 7639328 PMC1869811

[ref11] Eng M. L. Welty T. E. (2010). Management of hallucinations and psychosis in Parkinson's disease. Am. J. Geriatr. Pharmacother. 8, 316–330. doi: 10.1016/j.amjopharm.2010.08.004 20869621

[ref12] Factor S. A. Molho E. S. Podskalny G. D. Brown D. (1995). Parkinson's disease: drug-induced psychiatric states. Adv. Neurol. 65, 115–138. PMID: 7872135

[ref13] Fleitas C. Piñol-Ripoll G. Marfull P. Rocandio D. Ferrer I. Rampon C.. (2018). proBDNF is modified by advanced glycation end products in Alzheimer's disease and causes neuronal apoptosis by inducing p75 neurotrophin receptor processing. Mol. Brain 11:68. doi: 10.1186/s13041-018-0411-6, PMID: 30428894 PMC6237019

[ref14] Forsaa E. B. Larsen J. P. Wentzel-Larsen T. Goetz C. G. Stebbins G. T. Aarsland D.. (2010). A 12-year population-based study of psychosis in Parkinson disease. Arch. Neurol. 67, 996–1001. doi: 10.1001/archneurol.2010.166, PMID: 20697051

[ref15] Friedman J. H. (2010). Parkinson's disease psychosis 2010: a review article. Parkinsonism Relat. Disord. 16, 553–560. doi: 10.1016/j.parkreldis.2010.05.004 20538500

[ref16] Fujimura H. Altar C. A. Chen R. Nakamura T. Nakahashi T. Kambayashi J.. (2002). Brain-derived neurotrophic factor is stored in human platelets and released by agonist stimulation. Thromb. Haemost. 87, 728–734. doi: 10.1055/s-0037-1613072, PMID: 12008958

[ref17] Gejl A. K. Enevold C. Bugge A. Andersen M. S. Nielsen C. H. Andersen L. B. (2019). Associations between serum and plasma brain-derived neurotrophic factor and influence of storage time and centrifugation strategy. Sci. Rep. 9:9655. doi: 10.1038/s41598-019-45976-5 31273250 PMC6609657

[ref18] Goetz C. G. Stebbins G. T. (1993). Risk factors for nursing home placement in advanced Parkinson's disease. Neurology 43, 2227–2229. doi: 10.1212/wnl.43.11.2227, PMID: 8232934

[ref19] Hacksell U. Burstein E. S. McFarland K. Mills R. G. Williams H. (2014). On the discovery and development of pimavanserin: a novel drug candidate for Parkinson's psychosis. Neurochem. Res. 39, 2008–2017. doi: 10.1007/s11064-014-1293-3, PMID: 24682754 PMC4172996

[ref20] Halberstadt A. L. Powell S. B. Geyer M. A. (2013). Role of the 5-HT(2)A receptor in the locomotor hyperactivity produced by phenylalkylamine hallucinogens in mice. Neuropharmacology 70, 218–227. doi: 10.1016/j.neuropharm.2013.01.014, PMID: 23376711 PMC3934507

[ref21] Henderson M. J. M. Mellers J. D. C. (2000). Psychosis in Parkinson’s disease: ‘between a rock and a hard place’. Int. Rev. Psychiatry 12, 319–334. doi: 10.1080/09540260020002541

[ref22] Hernandez-Chan N. G. Bannon M. J. Orozco-Barrios C. E. Escobedo L. Zamudio S. De la Cruz F.. (2015). Neurotensin-polyplex-mediated brain-derived neurotrophic factor gene delivery into nigral dopamine neurons prevents nigrostriatal degeneration in a rat model of early Parkinson's disease. J. Biomed. Sci. 22:59. doi: 10.1186/s12929-015-0166-7, PMID: 26198255 PMC4511027

[ref23] Jiang H. Liu Y. Zhang Y. Chen Z. Y. (2011). Association of plasma brain-derived neurotrophic factor and cardiovascular risk factors and prognosis in angina pectoris. Biochem. Biophys. Res. Commun. 415, 99–103. doi: 10.1016/j.bbrc.2011.10.020, PMID: 22020095

[ref24] Johnson M. E. Lim Y. Senthilkumaran M. Zhou X. F. Bobrovskaya L. (2015). Investigation of tyrosine hydroxylase and BDNF in a low-dose rotenone model of Parkinson's disease. J. Chem. Neuroanat. 70, 33–41. doi: 10.1016/j.jchemneu.2015.11.002, PMID: 26562783

[ref25] Kapczinski F. Frey B. N. Kauer-Sant'Anna M. Grassi-Oliveira R. (2008). Brain-derived neurotrophic factor and neuroplasticity in bipolar disorder. Expert. Rev. Neurother. 8, 1101–1113. doi: 10.1586/14737175.8.7.1101 18590480

[ref26] Karege F. Schwald M. Cisse M. (2002). Postnatal developmental profile of brain-derived neurotrophic factor in rat brain and platelets. Neurosci. Lett. 328, 261–264. doi: 10.1016/s0304-3940(02)00529-3, PMID: 12147321

[ref27] Klein A. B. Williamson R. Santini M. A. Clemmensen C. Ettrup A. Rios M.. (2011). Blood BDNF concentrations reflect brain-tissue BDNF levels across species. Int. J. Neuropsychopharmacol. 14, 347–353. doi: 10.1017/S1461145710000738, PMID: 20604989

[ref28] Kowianski P. Lietzau G. Czuba E. Waskow M. Steliga A. Morys J. (2018). BDNF: A Key Factor with Multipotent Impact on Brain Signaling and Synaptic Plasticity. Cell. Mol. Neurobiol. 38, 579–593. doi: 10.1007/s10571-017-0510-4, PMID: 28623429 PMC5835061

[ref29] Larsen M. H. Hay-Schmidt A. Ronn L. C. Mikkelsen J. D. (2008). Temporal expression of brain-derived neurotrophic factor (BDNF) mRNA in the rat hippocampus after treatment with selective and mixed monoaminergic antidepressants. Eur. J. Pharmacol. 578, 114–122. doi: 10.1016/j.ejphar.2007.08.050, PMID: 17950272

[ref1001] Lavigne E. G. Buttigieg D. Steinschneider R. Burstein E. S. (2021). Pimavanserin Promotes Trophic Factor Release and Protects Cultured Primary Dopaminergic Neurons Exposed to MPP+ in a GDNF-Dependent Manner. ACS Chem. Neurosci. 12, 2088–2098. doi: 10.1021/acschemneuro.0c00751 34032411

[ref30] Lessmann V. Gottmann K. Malcangio M. (2003). Neurotrophin secretion: current facts and future prospects. Prog. Neurobiol. 69, 341–374. doi: 10.1016/s0301-0082(03)00019-4, PMID: 12787574

[ref31] Lommatzsch M. Zingler D. Schuhbaeck K. Schloetcke K. Zingler C. Schuff-Werner P.. (2005). The impact of age, weight and gender on BDNF levels in human platelets and plasma. Neurobiol. Aging 26, 115–123. doi: 10.1016/j.neurobiolaging.2004.03.002, PMID: 15585351

[ref32] Malin D. H. Tsai P. H. Campbell J. R. Moreno G. L. Chapman H. L. Suzaki A.. (2023). Pimavanserin reverses multiple measures of anxiety in a rodent model of post-traumatic stress disorder. Eur. J. Pharmacol. 939:175437. doi: 10.1016/j.ejphar.2022.175437, PMID: 36502961

[ref33] Marsh L. Williams J. R. Rocco M. Grill S. Munro C. Dawson T. M. (2004). Psychiatric comorbidities in patients with Parkinson disease and psychosis. Neurology 63, 293–300. doi: 10.1212/01.wnl.0000129843.15756.a3 15277623

[ref34] McFarland K. Price D. L. Bonhaus D. W. (2011). Pimavanserin, a 5-HT2A inverse agonist, reverses psychosis-like behaviors in a rodent model of Parkinson's disease. Behav. Pharmacol. 22, 681–692. doi: 10.1097/FBP.0b013e32834aff98, PMID: 21921840

[ref35] Meltzer H. Y. Mills R. Revell S. Williams H. Johnson A. Bahr D.. (2010). Pimavanserin, a serotonin(2A) receptor inverse agonist, for the treatment of parkinson's disease psychosis. Neuropsychopharmacology 35, 881–892. doi: 10.1038/npp.2009.176, PMID: 19907417 PMC3055369

[ref36] Minichiello L. Korte M. Wolfer D. Kuhn R. Unsicker K. Cestari V.. (1999). Essential role for TrkB receptors in hippocampus-mediated learning. Neuron 24, 401–414. doi: 10.1016/s0896-6273(00)80853-3, PMID: 10571233

[ref37] Mogi M. Nagatsu T. (1999). Neurotrophins and cytokines in Parkinson's disease. Adv. Neurol. 80, 135–139. PMID: 10410713

[ref38] Molteni R. Calabrese F. Bedogni F. Tongiorgi E. Fumagalli F. Racagni G.. (2006). Chronic treatment with fluoxetine up-regulates cellular BDNF mRNA expression in rat dopaminergic regions. Int. J. Neuropsychopharmacol. 9, 307–317. doi: 10.1017/S1461145705005766, PMID: 16035958

[ref39] Nagappan G. Zaitsev E. Senatorov V. V. Jr. Yang J. Hempstead B. L. Lu B. (2009). Control of extracellular cleavage of ProBDNF by high frequency neuronal activity. Proc. Natl. Acad. Sci. U. S. A. 106, 1267–1272. doi: 10.1073/pnas.0807322106, PMID: 19147841 PMC2633536

[ref40] Nakahashi T. Fujimura H. Altar C. A. Li J. Kambayashi J. Tandon N. N.. (2000). Vascular endothelial cells synthesize and secrete brain-derived neurotrophic factor. FEBS Lett. 470, 113–117. doi: 10.1016/s0014-5793(00)01302-8, PMID: 10734218

[ref41] Ondo W. G. Tintner R. Voung K. D. Lai D. Ringholz G. (2005). Double-blind, placebo-controlled, unforced titration parallel trial of quetiapine for dopaminergic-induced hallucinations in Parkinson's disease. Mov. Disord. 20, 958–963. doi: 10.1002/mds.20474, PMID: 15800937

[ref42] Pan W. Banks W. A. Fasold M. B. Bluth J. Kastin A. J. (1998). Transport of brain-derived neurotrophic factor across the blood-brain barrier. Neuropharmacology 37, 1553–1561. doi: 10.1016/s0028-3908(98)00141-5 9886678

[ref43] Pang P. T. Teng H. K. Zaitsev E. Woo N. T. Sakata K. Zhen S.. (2004). Cleavage of proBDNF by tPA/plasmin is essential for long-term hippocampal plasticity. Science 306, 487–491. doi: 10.1126/science.1100135 15486301

[ref44] Parkinson Study G. (1999). Low-dose clozapine for the treatment of drug-induced psychosis in Parkinson's disease. N. Engl. J. Med. 340, 757–763. doi: 10.1056/NEJM199903113401003, PMID: 10072410

[ref45] Picconi B. Piccoli G. Calabresi P. (2012). Synaptic dysfunction in Parkinson's disease. Adv. Exp. Med. Biol. 970, 553–572. doi: 10.1007/978-3-7091-0932-8_24 22351072

[ref46] Pillai A. (2008). Brain-derived neurotropic factor/TrkB signaling in the pathogenesis and novel pharmacotherapy of schizophrenia. Neurosignals 16, 183–193. doi: 10.1159/000111562 18253057

[ref47] Pillai A. Kale A. Joshi S. Naphade N. Raju M. S. Nasrallah H.. (2010). Decreased BDNF levels in CSF of drug-naive first-episode psychotic subjects: correlation with plasma BDNF and psychopathology. Int. J. Neuropsychopharmacol. 13, 535–539. doi: 10.1017/S1461145709991015, PMID: 19941699

[ref48] Pillai A. Terry A. V. Jr. Mahadik S. P. (2006). Differential effects of long-term treatment with typical and atypical antipsychotics on NGF and BDNF levels in rat striatum and hippocampus. Schizophr. Res. 82, 95–106. doi: 10.1016/j.schres.2005.11.021, PMID: 16442781

[ref49] Poduslo J. F. Curran G. L. (1996). Permeability at the blood-brain and blood-nerve barriers of the neurotrophic factors: NGF, CNTF, NT-3, BDNF. Brain Res. Mol. Brain Res. 36, 280–286. doi: 10.1016/0169-328x(95)00250-v, PMID: 8965648

[ref50] Polyakova M. Schlogl H. Sacher J. Schmidt-Kassow M. Kaiser J. Stumvoll M.. (2017). Stability of BDNF in Human Samples Stored Up to 6 Months and Correlations of Serum and EDTA-Plasma Concentrations. Int. J. Mol. Sci. 18:1189. doi: 10.3390/ijms18061189, PMID: 28587193 PMC5486012

[ref51] Rabey J. M. Prokhorov T. Miniovitz A. Dobronevsky E. Klein C. (2007). Effect of quetiapine in psychotic Parkinson's disease patients: a double-blind labeled study of 3 months' duration. Mov. Disord. 22, 313–318. doi: 10.1002/mds.21116, PMID: 17034006

[ref52] Rizos E. N. Papathanasiou M. Michalopoulou P. G. Mazioti A. Douzenis A. Kastania A.. (2011). Association of serum BDNF levels with hippocampal volumes in first psychotic episode drug-naive schizophrenic patients. Schizophr. Res. 129, 201–204. doi: 10.1016/j.schres.2011.03.011, PMID: 21470828

[ref53] Rogoz Z. Skuza G. Legutko B. (2008). Repeated co-treatment with fluoxetine and amantadine induces brain-derived neurotrophic factor gene expression in rats. Pharmacol. Rep. 60, 817–826. PMID: 19211973

[ref54] Serra-Millas M. (2016). Are the changes in the peripheral brain-derived neurotrophic factor levels due to platelet activation? World J. Psychiatry. 6, 84–101. doi: 10.5498/wjp.v6.i1.84, PMID: 27014600 PMC4804271

[ref55] Shi Y. You J. Yuan Y. Zhang X. Li H. Hou G. (2010). Plasma BDNF and tPA are associated with late-onset geriatric depression. Psychiatry Clin. Neurosci. 64, 249–254. doi: 10.1111/j.1440-1819.2010.02074.x, PMID: 20602725

[ref56] Shotbolt P. Samuel M. Fox C. David A. S. (2009). A randomized controlled trial of quetiapine for psychosis in Parkinson's disease. Neuropsychiatr. Dis. Treat. 5, 327–332. doi: 10.2147/ndt.s5335, PMID: 19557142 PMC2699657

[ref57] Tang M. Jiang P. Li H. Cai H. Liu Y. Gong H.. (2015). Antidepressant-like effect of n-3 PUFAs in CUMS rats: role of tPA/PAI-1 system. Physiol. Behav. 139, 210–215. doi: 10.1016/j.physbeh.2014.11.054 25449400

[ref58] Teng H. K. Teng K. K. Lee R. Wright S. Tevar S. Almeida R. D.. (2005). ProBDNF induces neuronal apoptosis via activation of a receptor complex of p75NTR and sortilin. J. Neurosci. 25, 5455–5463. doi: 10.1523/JNEUROSCI.5123-04.2005 15930396 PMC6724992

[ref59] The French Clozapine Parkinson Study, G. (1999). Clozapine in drug-induced psychosis in Parkinson's disease. The French Clozapine Parkinson Study Group. Lancet 353, 2041–2042. 10376627

[ref60] Theilmann W. Rosenholm M. Hampel P. Loscher W. Rantamaki T. (2020). Lack of antidepressant effects of burst-suppressing isoflurane anesthesia in adult male Wistar outbred rats subjected to chronic mild stress. PLoS One 15:e0235046. doi: 10.1371/journal.pone.0235046, PMID: 32579566 PMC7313995

[ref61] Vaidya V. A. Marek G. J. Aghajanian G. K. Duman R. S. (1997). 5-HT2A receptor-mediated regulation of brain-derived neurotrophic factor mRNA in the hippocampus and the neocortex. J. Neurosci. 17, 2785–2795. doi: 10.1523/JNEUROSCI.17-08-02785.1997, PMID: 9092600 PMC6573109

[ref62] Vanover K. E. Weiner D. M. Makhay M. Veinbergs I. Gardell L. R. Lameh J.. (2006). Pharmacological and behavioral profile of N-(4-fluorophenylmethyl)-N-(1-methylpiperidin-4-yl)-N'-(4-(2-methylpropyloxy)phenylmethyl) carbamide (2R,3R)-dihydroxybutanedioate (2:1) (ACP-103), a novel 5-hydroxytryptamine(2A) receptor inverse agonist. J. Pharmacol. Exp. Ther. 317, 910–918. doi: 10.1124/jpet.105.097006, PMID: 16469866

[ref63] Wang Y. Liu H. Zhang B. S. Soares J. C. Zhang X. Y. (2016). Low BDNF is associated with cognitive impairments in patients with Parkinson's disease. Parkinsonism Relat. Disord. 29, 66–71. doi: 10.1016/j.parkreldis.2016.05.023, PMID: 27245919

[ref64] Wang M. Xie Y. Qin D. (2021). Proteolytic cleavage of proBDNF to mBDNF in neuropsychiatric and neurodegenerative diseases. Brain Res. Bull. 166, 172–184. doi: 10.1016/j.brainresbull.2020.11.005, PMID: 33202257

[ref65] Xu B. Gottschalk W. Chow A. Wilson R. I. Schnell E. Zang K.. (2000). The role of brain-derived neurotrophic factor receptors in the mature hippocampus: modulation of long-term potentiation through a presynaptic mechanism involving TrkB. J. Neurosci. 20, 6888–6897. doi: 10.1523/JNEUROSCI.20-18-06888.2000, PMID: 10995833 PMC2711895

[ref66] Zhang S. S. Tian Y. H. Jin S. J. Wang W. C. Zhao J. X. Si X. M.. (2019). Isoflurane produces antidepressant effects inducing BDNF-TrkB signaling in CUMS mice. Psychopharmacology 236, 3301–3315. doi: 10.1007/s00213-019-05287-z, PMID: 31197433

[ref67] Zhang F. Zhu Z. Q. Liu D. X. Zhang C. Gong Q. H. Zhu Y. H. (2014). Emulsified isoflurane anesthesia decreases brain-derived neurotrophic factor expression and induces cognitive dysfunction in adult rats. Exp. Ther. Med. 8, 471–477. doi: 10.3892/etm.2014.1769, PMID: 25009603 PMC4079394

[ref68] Zhou L. Xiong J. Lim Y. Ruan Y. Huang C. Zhu Y.. (2013). Upregulation of blood proBDNF and its receptors in major depression. J. Affect. Disord. 150, 776–784. doi: 10.1016/j.jad.2013.03.002, PMID: 23537780

